# Crystal Structural Characteristics and Electrical Properties of Novel Sol-Gel Synthesis of Ceramic Bi_0.75_Ba_0.25_(FeMn)_0.5_O_3_

**DOI:** 10.3390/ma17153797

**Published:** 2024-08-01

**Authors:** Faouzia Tayari, Ramzi Dhahri, Elkenany Brens Elkenany, Sílvia Soreto Teixeira, Manuel Pedro Fernandes Graça, A. M. Al-Syadi, Manel Essid, Kais Iben Nassar

**Affiliations:** 1 i3N-Physics Department, University of Aveiro, 3810-193 Aveiro, Portugal; faouziatayari12@gmail.com (F.T.); silvia.soreto@ua.pt (S.S.T.); mpfg@ua.pt (M.P.F.G.); maiseed2175@gmail.com (M.E.); 2 Department of Physics, College of Science and Arts, Najran University, Najran 11001, Saudi Arabia; ebabdelmoaty@nu.edu.sa (E.B.E.); amalsyadi@nu.edu.sa (A.M.A.-S.); 3 CICECO—Aveiro Institute of Materials, Department of Chemistry, University of Aveiro, Campus Universitário de Santiago, 3810-193 Aveiro, Portugal

**Keywords:** perovskite, sol-gel route, X-ray diffraction, impedance, dielectric, conductivity

## Abstract

In this investigation, our primary objective is to explore the structural, morphological, and electrical characteristics of Bi_0.75_Ba_0.25_(FeMn)_0.5_O_3_ ceramic material synthesized by the sol-gel method. The prepared sample underwent synthesis through the conventional sol-gel technique. Examination through X-ray diffraction (XRD) unveiled a well-defined rhombohedral structure within the R$\overset{´}{3}$C space group. Moreover, to evaluate the purity and nano-grain morphology, we utilized energy dispersive spectroscopy (EDX) and scanning electron microscopy (SEM). Electrical assessments were carried out over a frequency span of 100 Hz to 1 MHz and temperatures ranging from 200 to 340 K. Employing the correlated barrier hopping (CBH) model, we analyzed the AC conductivity of our specimen. The activation energy, determined from both DC conductivity and impedance spectra, demonstrated close correspondence, suggesting that both conductivity and r laxation processes are influenced by similar factors. Notably, the dielectric properties hold significant importance, potentially rendering our sample suitable for electronic applications. Furthermore, we calculated thermodynamic parameters, such as enthalpy (ΔH), entropy change (ΔS), and free energy of activation (ΔF), offering deeper insights into the material’s behavior and conductivity mechanisms.

## 1. Introduction

The challenge of data storage has persisted since the early 20th century, accentuated by technological advancements and the escalating computing power. Storage memories have progressively become ubiquitous in everyday devices. Over time, the pursuit has been to augment storage capacity while concurrently reducing access times for reading and encoding information. With the ongoing trend in electronics towards miniaturization and enhanced speed, there’s a pressing need for alternatives to current materials. This has led researchers to explore new avenues, seeking multifunctional materials capable of outperforming existing monofunctional ones while ensuring greater efficiency. The focus has particularly shifted towards double perovskite materials, drawn by their remarkable properties and the potential for innovative technological applications, especially in the realm of non-volatile memory design. Perovskite compounds exhibit a general formula of AB′B″O_3_ or A′A″B′B″O_3_, wherein the A site is primarily occupied by alkaline earth or rare earth metal ions, while the B’ and B’’ sites accommodate two transition metals, forming octahedra denoted as B’O_6_ and B’’O_6_. These compounds have garnered significant attention in recent years due to their diverse physical properties. Moreover, they are characterized by strong correlations among their structural, electrical, dielectric, optical, and magnetic features. The intriguing aspect lies in the variation of B′ and B″ cations, offering a broad spectrum of electrical and magnetic properties. These include metallicity, semi-metallicity, insulation, as well as distinct magnetic behaviors such as ferromagnetism (FM), ferrimagnetism (FIM), and antiferromagnetism (AFM) [1,2]. On the other hand, perovskite materials are inherently multiferroic, a property recognized since 1960. These materials exhibit at least two distinct ferroic orders (ferromagnetic and ferroelectric) within the same temperature range, making them subjects of considerable interest in both fundamental physics and chemistry [3,4,5]. In the realm of ferroelectricity, the term ‘ferro’ signifies the presence of a permanent electric polarization even in the absence of an electric field, while in ferromagnetism, there is a residual magnetization reversible by the application of a magnetic field. The initial investigations focused on oxides of the Ln_1−x_A_x_Mn$O_{3}$ type, where x ∈ [0, 1] (Ln = rare earth, A = Ca, Ba, Sr, …), marking the inception of research in this area [6,7]. Manganese, a constituent of these oxides, exhibits crucial magnetic properties, including both ferromagnetic and antiferromagnetic behavior.

Furthermore, researchers have taken a keen interest in perovskite compounds owing to their diverse physical properties, which can be manipulated by the application of electric and magnetic fields. Ferroelectric perovskites stand out as particularly intriguing examples of such compounds, finding utility in a range of technological applications [8,9,10,11,12]. These applications encompass non-volatile memories, sensors, capacitors, high-voltage line insulation, electric motor spark plugs, circuit breakers and switches, as well as microwave dielectric filters and pyroelectric detectors [13,14]. The broad applicability of perovskites in modern electronics is attributed to their noteworthy attributes, including high dielectric permittivity, a substantial piezoelectric coefficient, ferroelectric behavior, semiconductivity, catalytic activity, and thermoelectric properties. Notably, these oxides exhibit a diverse array of crystalline structures. Around room temperature, they may assume a cubic structure (Pm-3m), exemplified by LaFeO_3_ [15], tetragonal configuration (I4/m), as seen in Ba_0.97_La_0.02_Ti_0.9_ Nb_0.08_O_3_ [16], and monoclinic structure (P21/n), illustrated by BaBiO_3_ [17].

In the literature, the customization of the physical properties of multiferroic BiFeO_3_ was undertaken by introducing NaTaO_3_ ferroelectric, resulting in the formation of (Bi_1−x_, Na_x_)(Fe_1−x_, Ta_x_)O_3_ (x = 0.0, 0.1, 0.2, and 0.3) solid solutions. The confirmation of the desired materials was achieved through X-ray diffraction analysis [18]. Examination of the surface texture via SEM revealed a uniform grain distribution with a few small voids. The impedance and dielectric properties of these materials were investigated across different concentrations (x) of NaTaO_3_, considering variations in temperature and frequency. Of particular significance were the relative dielectric constant and loss tangent. In our specific case, we studied Bi_0.75_Ba_0.25_(FeMn)_0.5_O_3_ ceramic to enhance its electrical and dielectric properties, employing complex impedance spectroscopy for electric devices. This study primarily aims to fabricate this polycrystalline material through the sol-gel route and conduct a comprehensive analysis of its structural, morphological, and electrical attributes. Techniques such as X-ray diffraction and scanning electron microscopy were employed. Additionally, we examined the dielectric relaxation and conductivity as functions of frequency and temperature, with an eye toward potential applications in electrical devices.

## 2. Experimental Details

### 2.1. Sample Preparation

The Bi_0.75_Ba_0.25_(FeMn)_0.5_O_3_ sample was synthesized via the sol–gel method.

Initially, metal precursors—Bismuth Oxide (Bi_2_O_3_) in the amount of 0.75 moles (349.47 g), Barium Carbonate (BaCO_3_ in 0.25 moles (34.33 g), Iron Oxide (Fe_2_O_3_) in 0.25 moles (39.92 g), and Manganese Oxide (Mn_2_O_3_) in 0.25 moles (57.20 g)—were dissolved in nitric acid to form metal nitrate solutions. Distilled water was added as needed to achieve a clear solution with the desired metal concentrations. The solution was then heated at 80 °C for 5 h with continuous stirring using a magnetic mixer to ensure complete dissolution and uniform mixing. Citric acid (1.5 moles, 288.20 g) and ethylene glycol (1.0 moles, 62.07 g) were added to the solution in a 1:1 molar ratio relative to the metal ions to facilitate gel formation. The resulting solution was allowed to gel and then dried to remove water, forming a solid gel. The dried gel was subsequently calcined at an appropriate temperature to obtain the final Bi_0.75_Ba_0.25_(FeMn)_0.5_O_3_ ceramic material. These quantities and steps were optimized to ensure the correct stoichiometry and phase formation of the ceramic.

The resulting mixture underwent drying and calcination processes to yield a nano-crystalline powder. This powder was then crushed, pressed into a pellet, and subjected to sintering. Finally, the material underwent annealing in the temperature range of 550 °C to 1100 °C for duration of 12 h to achieve homogeneity. The material underwent a carefully controlled annealing process in the temperature range of 550 °C to 1100 °C. This temperature scheme was chosen based on preliminary optimization experiments aimed at achieving the best crystallinity and phase purity of the prepared sample. The lower bound of 550 °C was selected to initiate grain growth and remove any residual stresses, while the upper bound of 1100 °C was determined to be the maximum temperature at which the material could be annealed without causing decomposition or undesirable phase transitions. In addition, the optimization involved systematic variations in the annealing temperature and duration, with X-ray diffraction (XRD) and scanning electron microscopy (SEM) analyses conducted to monitor phase purity, crystallite size, and microstructural evolution. The chosen temperature range represents the optimal conditions that resulted in the highest quality material with the desired properties for our study.

The compound was synthesized according to the following equation:

Bi_2_O_3_ + BaCO_3_ + Fe_2_O_3_ + Mn_2_O_3_ ==========> Bi_0.75_Ba_0.25_(FeMn)_0.5_O_3_ + CO_2_ + O_2_

### 2.2. Sample Characterization

The powder underwent characterization through X-ray diffraction (XRD) using an X’Pert MPD Philips diffractometer (Philips, Amsterdam, The Netherlands) at the University of Aveiro, Portugal, employing CuKα radiation with a wavelength (λ) of 1.54060 Å, and the analysis was conducted at room temperature. Surface morphology analysis was performed using scanning electron microscopy (SEM) with a TESCAN Vega 3 microscope (TESCAN, Brno, Czech Republic) at the University of Aveiro, Portugal. Prior to microscopic observation, the sample underwent carbon coating to enhance surface conductivity. For electrical and dielectric measurements, the complex impedance spectroscopy (IS) technique was employed. This technique typically involves cells with two identical electrodes applied to the faces of a sample. The measurements were conducted using a precision impedance analyzer (Agilent 4294A, Agilent Technologies, Santa Clara, CA, USA) at the University of Aveiro, Portugal, in a Cp-Rp configuration (capacitance in parallel with resistance). The frequency range spanned from 100 Hz to 1 MHz, and the temperature range was 200 to 340 K. A cryostat-bath, operating in a helium atmosphere, was utilized to optimize heat transfer and prevent humidity interference. To facilitate electrical measurements, the compound was prepared in the form of a metallized disk, configured as a planar capacitor. In IS, various electrical stimuli can be used, with the most common being the measurement of impedance directly in the frequency domain by applying an alternating voltage. In this study, the sintered pellets, processed at a temperature of 1100 °C for electrical measurements, were coated with silver paste to ensure robust ohmic contacts. Also, we have conducted current-voltage (I–V) measurements to confirm the ohmic nature of the contacts made with silver paste. The I–V characteristics demonstrated a linear relationship, verifying that the silver paste provided robust ohmic contacts. This ensures that the electrical measurements obtained are accurate and not influenced by contact resistance. The samples used in this study were prepared with specific dimensions to ensure consistent and accurate measurements. For electrical characterizations, a disk-shaped sample with a diameter of 11.37 mm and a thickness of approximately 1.52 mm was utilized.

## 3. Results and Discussion

### 3.1. Phase Identification and Crystal Structure

**Figure 1:**
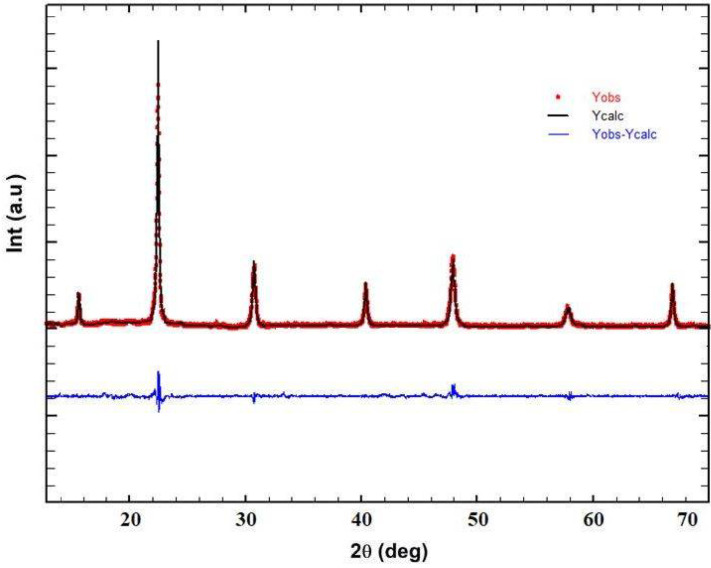
X-ray diffraction (XRD) of Bi_0.75_Ba_0.25_(FeMn)_0.5_O_3_ at room temperature.

Figure 1 illustrates the X-ray diffraction (XRD) pattern, revealing the absence of secondary phases. This observation indicates that the material is a single-phase and well-crystallized substance. The crystal structure belongs to the rhombohedral system with space group R$\overset{´}{3}$C, and the agreement between calculated and measured intensities attests to the high level of crystallization. Refinement results provide crucial information such as crystal structure parameters, lattice constants, atomic coordinates, and mean bond lengths. The refined structural parameters, including angles α, β, γ, as well as reliability factors (*X*^2^), Bragg factor (R_B_), and bond lengths for the prepared sample, are summarized in Table 1. Based on our X-ray diffraction results, the phase identification of our sample matches the perovskite-like structure with the rhombohedral crystal system, space group R$\overset{´}{3}$C. For accurate reference and comparison, we include the JCPDS card number [JCPDS No. 00-027-0086], which corresponds to the standard pattern for a similar rhombohedral perovskite phase.

To further affirm the presence of the perovskite-like structure and assess the degree of distortion in this compound, we calculated the Goldschmidt tolerance factor (t) [18] using the formula:

(1) $$t=\frac{r_{A}+r_{O}}{\sqrt{2}\left(r|B+r_{O}\right)}$$

Here, *r_A_* = *r* (Ba and Bi), *r_B_* = *r* (Fe and Mn), and *r_O_* is the ionic radii associated with the cations of sites *A*, *B*, and oxygen, respectively. The evolution of crystal structures as a function of the tolerance factor is explained below: 0.75 < t < 0.96: orthorhombic distortion; 0.96 < t < 0.99: rhombohedral distortion; and 0.99 < t < 1.06: cubic. According to Shannon, the ionic radii are as follows: (rBi^3+^) = 1.17 Ǻ, (rBa^2+^) =1.35 Å, (rFe^2+^) = 0.49 Ǻ, (rFe^3+^) = 0.585 Ǻ, (rMn^4+^) = 0.75 Ǻ, (rMn^3+^) = 0.58 Ǻ, and (rO^2−^) = 1.4 Ǻ [19,20]. For this material, it is less than 1, where t = 0.968. This confirms the rhombohedral system of the sample. In the context of the morphological study of grains, the mean crystallite size was determined utilizing the Debye–Scherrer formula [21]:

(2) $$D=\frac{0.9.\lambda }{\beta .cos\theta }$$

In this calculation, where D represents the average size of crystallites, *λ* is the wavelength (1.54060 Å), *β* is the width at mid-height of the peak, and *θ* is the diffraction angle of the considered peak. The determined grain size is 31 nm.

### 3.2. Morphological Analysis

**Figure 2:**
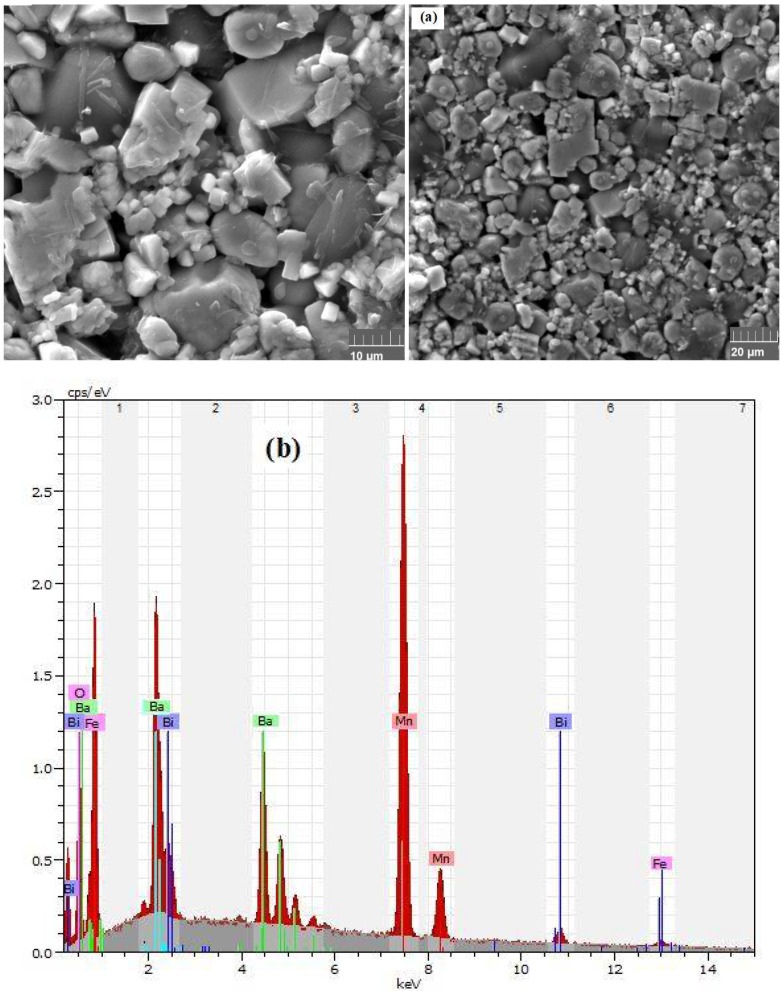
SEM images (**a**) and spectra obtained by EDS analysis (**b**) of Bi_0.75_Ba_0.25_(FeMn)_0.5_O_3_.

The scanning electron microscope is a well-established tool for assessing the homogeneity, morphology, and porosity of ceramics under study. Leveraging the principles of electron-matter interactions, SEM enables the generation of high-resolution images that capture the surface state of the sample. In our investigation, the morphology and grain size of the compound were scrutinized using a scanning electron microscope. In Figure 2a, micrographs of the sample captured at various scales are presented. The nature and size of the microstructures suggest a remarkably dense surface, attributed to the uniform distribution of grains. Notably, the observed grain size is 246 nm, exceeding the particle size calculated from the XRD results. This discrepancy is attributed to the fact that each particle identified by SEM comprises a collection of crystallites, potentially influenced by internal interactions or defects such as vacancies and dislocations. The determination of crystallite/grain size involved the use of image-J software (version 1.54j), specifically designed for calculating the average size of nanoparticles. The methodology entails measuring the sizes of individual particles from the SEM image, revealing variations in size. Qualitative analysis of the elements constituting the material was conducted through Energy Dispersive Spectrometry. This technique involves exciting a point on the sample with an electron beam, causing the emission of X-rays, which are subsequently analyzed. Chemical analysis by microanalysis (Figure 2b) was performed at multiple points across various samples. The spectra obtained from microanalysis clearly indicate the presence of all the chemical elements introduced during the elaboration process.

### 3.3. Impedance Spectroscopy

#### 3.3.1. Cole-Cole Plots

**Figure 3:**
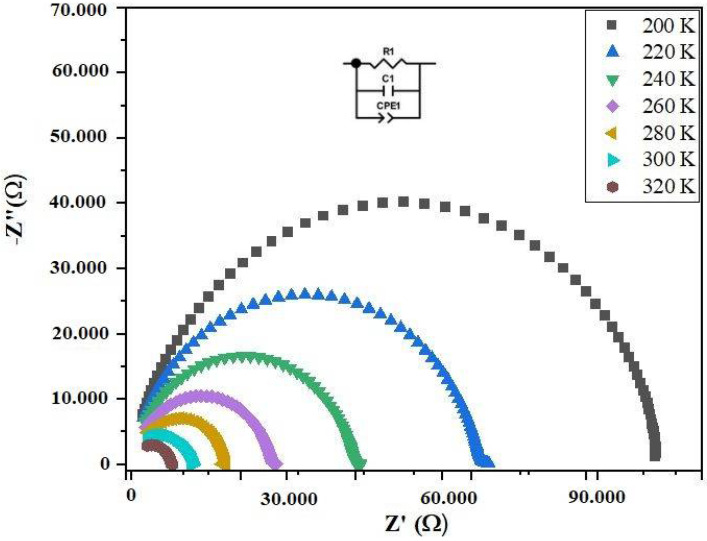
Nyquist plots of the Bi_0.75_Ba_0.25_(FeMn)_0.5_O_3_ with insert circuit equivalent.

The method of complex impedance spectroscopy (CIS) has been employed to differentiate the electrical characteristics of materials and their interactions with electronically conductive electrodes. The impedance data often converge towards each other. This behavior may be attributed to the release of space charges and a decrease in barrier properties, leading to an improvement in AC conductivity. Indeed, the Nyquist diagram serves as a tool to assess the contributions of grains and grain boundaries to conduction and to model the sample using an equivalent electrical circuit. Figure 3 illustrates the Nyquist plot (Z″ vs. Z′) for the ceramic prepared at various temperatures. The spectra in this graph feature semicircular arcs centered on the real axis Z′. Notably, the radius of these semicircles diminishes with increasing temperature, confirming the thermal activation of conduction processes and showcasing the semiconductor behavior of the material. It is important to highlight that these arcs exclusively represent the grain. A distinctive characteristic is observed in the form of non-Debye type relaxation [22,23].

Furthermore, the decrease in the diameter of the semicircular arcs with increasing temperature aligns with the semiconductor behavior of this material [23]. The impedance decrease with rising temperature signifies the displacement of charge carriers involved in the conduction mechanism [22]. To refine the different spectra, the Zview software (ZView version 3.4), is employed. Consequently, the experimental data for the compound are modeled using an equivalent circuit comprising three elements (R, C, and CPE) in parallel. The constant phase element (*Z_CPE_*) is determined using the following equation [24]:

(3) $$Z_{\mathrm{CPE}}=\frac{1}{Q{\left(jw\right)}^{\alpha }}$$

Alternatively, a fractal capacitance (CPE) was employed instead of a pure capacitance to accommodate the decentering of the associated semicircle from the *Z′* axis [25]. In a broader context, the utilization of a *CPE* reflects deviations from ideal dielectric behavior and, consequently, from a time distribution. The fractal capacitance is characterized by two parameters: a coefficient *Q* and an exponent α (0 ≤ *α* ≤ 1), representing the extent of deviation from an ideal system. The parameters of the equivalent electric circuit corresponding to the compound under study are derived from a well-fitted analysis and are summarized in Table 2.

**Figure 4:**
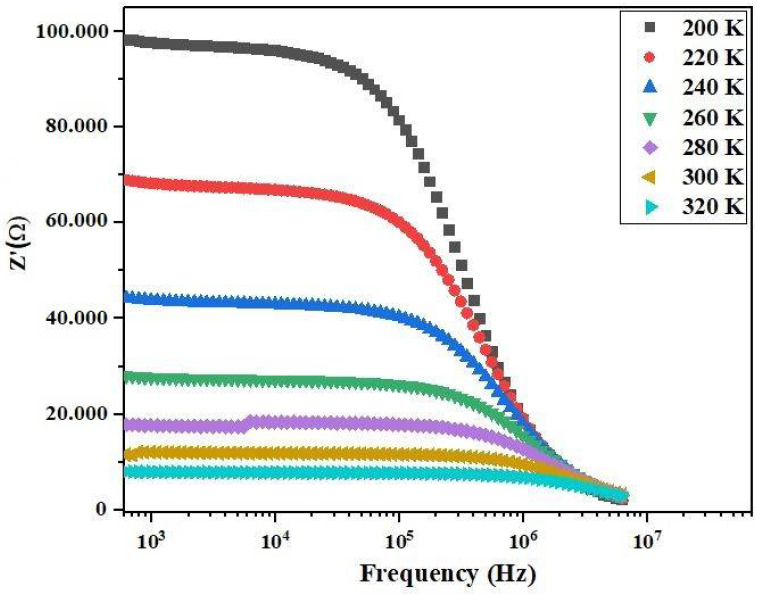
Frequency dependence of Z′(ω) at various temperatures of Bi_0.75_Ba_0.25_(FeMn)_0.5_O_3_ compound.

Figure 4 illustrates the frequency dependence of the real part of impedance (*Z*′) at different temperatures. Notably, at low frequencies, *Z*′ is higher at lower temperatures compared to higher temperatures, suggesting an increase in conductivity with rising temperature. However, at higher frequencies beyond 10^5^ Hz, these values gradually decrease and converge, potentially owing to the diminishing barrier at elevated temperatures, compensated by the release of space charges [22,23].

**Figure 5:**
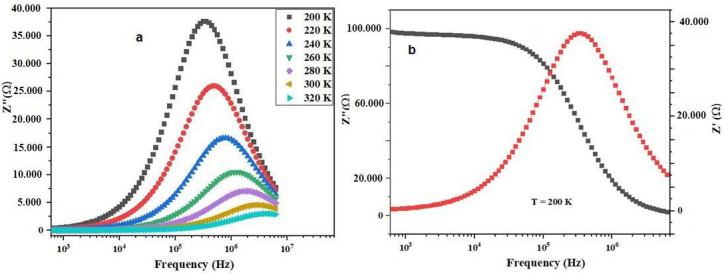
Frequency dependence of *Z″* (ω) at various temperatures of Bi_0.75_Ba_0.25_(FeMn)_0.5_O_3_ compound.

In Figure 5a, the variation in the imaginary part of the impedance (Z″) at different temperatures is depicted. It is evident that Z″ decreases with increasing frequency and rising temperature. Z″ attains a maximum at a specific frequency (Z_max_), attributed to the relaxation of the material [22]. The presence of relaxation phenomena are confirmed in the prepared ceramic. In Figure 5b, the variation of both the imaginary part (Z″) and the real part (Z′) of the impedance at 200 K as a function of frequency is illustrated. It can be observed that Z’ decreases steadily with increasing frequency, indicating a decrease in resistive behavior. Meanwhile, Z″ initially increases, reaches a peak (indicating a relaxation process), and then decreases, further confirming the dielectric relaxation phenomena in the ceramic material.

#### 3.3.2. DC Conductivity

**Figure 6:**
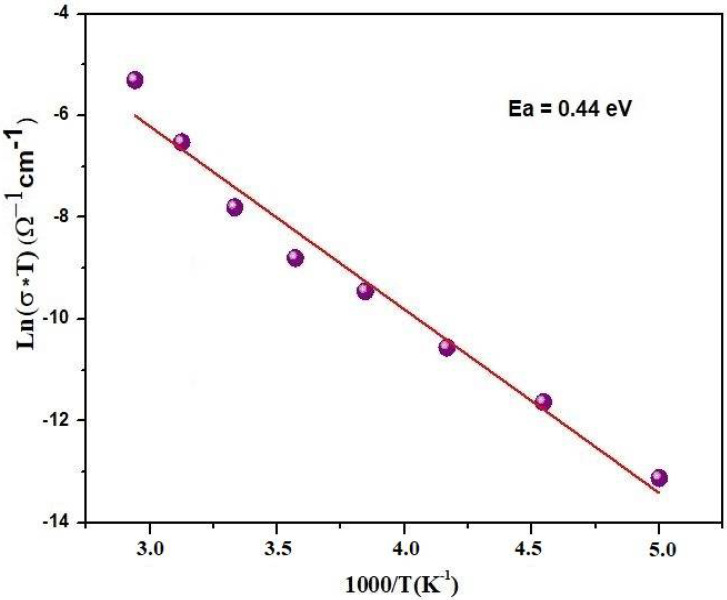
Arrhenius relation of Ln (σ_DC_*T) versus 1000/T for Bi_0.75_Ba_0.25_(FeMn)_0.5_O_3_.

The direct current ($\sigma_{DC}$) conductivity is associated with the terms of resistance (R). It can be expressed by the following equation:

(4) $$\sigma_{DC}=e/RT$$

where e represents the thickness and denotes the area of the compound. The Ln ($\sigma_{DC}$ × T), depicted as a function of 1000/T in Figure 6 from the complex impedance plots, exhibits an Arrhenius-type behavior defined by the following formula [26]:

(5) $$\sigma \left(T\right)=\sigma_{0}\;exp\left(\frac{-E_{a}}{K_{B}T}\right)$$

where *σ(T)* is the temperature-dependent conductivity, *σ*_0_ is a pre-exponential factor, *E_a_* is the activation energy, K_B_ is the Boltzmann constant, and T is the temperature, the plot reveals a consistent single slope across the examined temperature range. This uniform slope serves as an additional indication of the activation of a solitary conduction mechanism.

The activation energy, derived from the gradient of the *Ln* ($\sigma_{DC}$.*T*) versus 1000/T curve, is estimated to be approximately, *E_a_* = 0.44 eV. Moreover, this specific frequency is identified as the maximum relaxation frequency, denoted as *f_max_*, while its reciprocal is termed the relaxation time (*τ*). The shifts of the relaxation peaks towards the high-frequency region signify a reduction in the relaxation time with escalating temperature, as illustrated in Figure 5a. Furthermore, this relaxation phenomenon is presumably linked to defects at elevated temperatures and stationary species at lower temperatures. The activation energy for conductivity (*Ea*) is primarily influenced by the free carrier density. This is because the conduction process in materials often depends on the availability of free carriers, which requires energy to be excited from their bound states to the conduction band. The derived relaxation time (*τ*) is linked to the temperature dependence of carrier mobility or the diffusion coefficient. As the temperature increases, the mobility of carriers can change due to scattering mechanisms, which affects the relaxation time. This temperature dependence can be expressed using the Arrhenius-type equation for *τ*.

**Figure 7:**
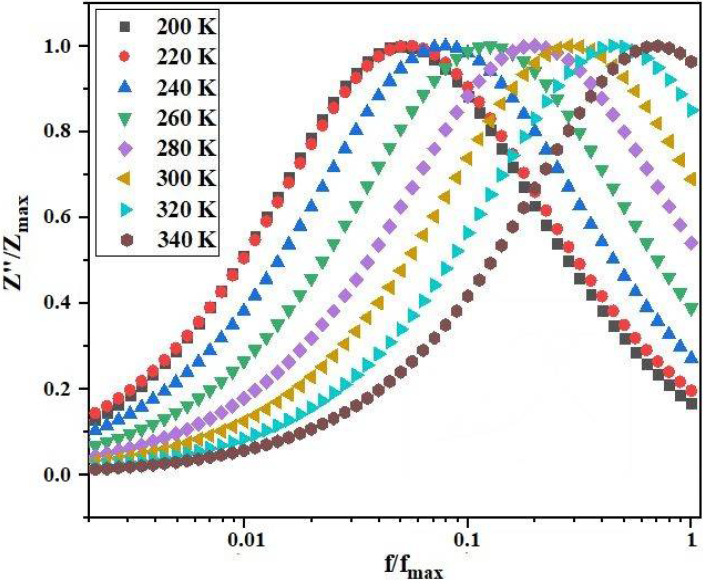
The normalized spectra of the imaginary impedance Z″/Z_max_ at different temperatures.

Additionally, the relaxation time conforms to Arrhenius’ law, as expressed by the following equation:

(6) $$\tau =\tau_{0}\;exp\left(\frac{-E_{\tau }}{K_{B}T}\right)$$

where *τ(T)* is the temperature-dependent relaxation time, *τ_0_* is a pre-exponential factor, *E_τ_* is the activation energy for relaxation, *K_B_* is the Boltzmann constant, and T is the temperature. The activation energy is defined by the energy required for the charge carrier to jump between neighboring sites [26]. We obtain as τ_0_ = 2.47 *×* 10^−10^ s. To explain the dielectric relaxation of the material, we plot the normalized spectrum of the imaginary Z″/Z_max_ part of the impedance a function of *f/f_max_* in Figure 7. None of the normalized spectra overlap, indicating that the dielectric relaxation is temperature dependent [27]. The evolution of *Z″/Z″_max_* at different temperatures shows the shift of peaks towards high frequencies.

#### 3.3.3. AC Conductivity

To scrutinize the dynamic response of the material concerning the applied electric field, the experimental data were subjected to an analysis of the conductivity, specifically termed alternating current conductivity (*σ_AC_*). This type of conductivity is linked to the conduction of electric charges aligned with the applied electric field, and its frequency dependence offers insights into the nature of the charge carriers. The frequency dependence of the conductivity adheres to Jonscher’s power law [28]:

(7) $$\sigma AC\left(\omega \right)=\sigma_{DC}+A\;\omega^{s}$$

**Figure 8:**
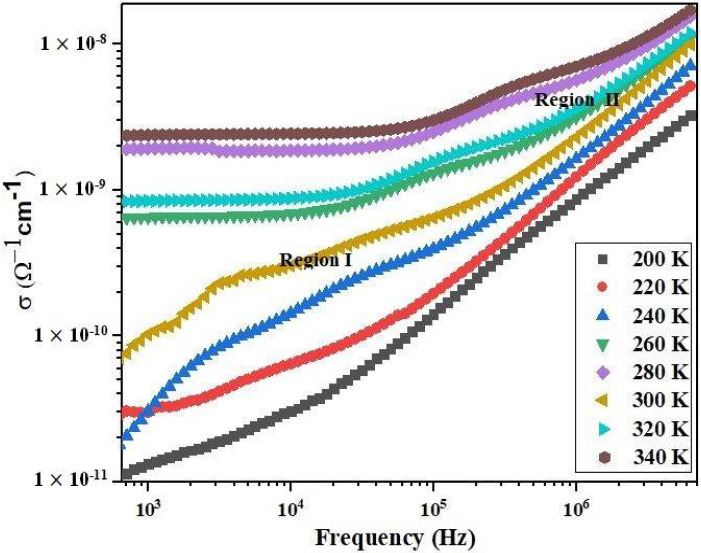
Frequency dependence of the AC conductivity (*σ_AC_*) measured using impedance spectroscopy.

Here, *σ_DC_* represents the direct current conductivity at low frequencies, while A serves as a coefficient determining the extent of polarizability [29], *ω* denotes the angular frequency, and S signifies the degree of interaction between mobile ions and the networks surrounding them. Figure 8 indicates the independent frequency and temperature variations of conductivity for the studied sample. It can be observed that below 10^5^ Hz, the AC conductivity remains frequency-independent for all temperatures (region I). Within this range, the AC conductivity closely mirrors the DC conductivity of the sample. Conversely, in the high-frequency region (region II), *σ_AC_* escalates with both frequency and temperature, a behavior typical of semiconductor materials. In accordance with Funke [30], two conditions can be elucidated: If *S* < 1, the electron jump is associated with a sudden displacement. According to Jonscher, the variation in high-frequency conductivity underlies the relaxation phenomenon in the material, driven by the mobility of the charge carriers, specifically their displacement to a new site from the original position. Figure 8 features a fit using Equation (7) with ORIGIN 8.0 software, and the experimental results are summarized in Table 3. This outcome suggests that the material is a semiconductor. Consequently, error bars on the data points account for experimental uncertainties.

However, when *S* > 1, the charge carriers’ jumps take place between adjacent sites. Indeed, Equation (7) can be described by the Almond-West relation [31]:

(8) $$\sigma {\mathrm{AC}}_{\;}=\sigma_{DC}\;(1+(\frac{\omega }{{\omega_{h}}^{S}}))$$

where *ω_h_* denotes the jump frequency of the charge carriers, defined by the following formula:

(9) $$\omega_{h}=(\frac{\sigma_{DC}}{A^{\frac{1}{S}}})$$

**Figure 9:**
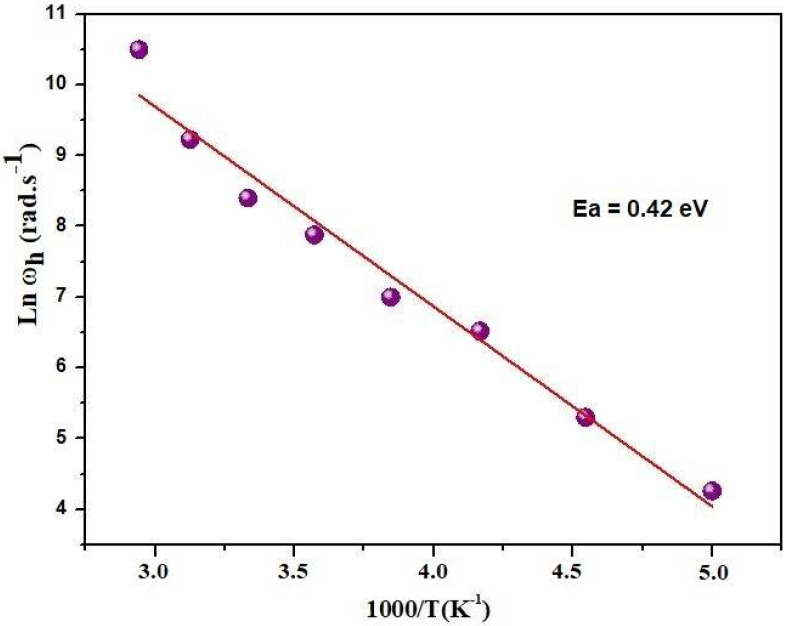
Variation of Ln (ω_h_) versus (1000/T).

Figure 9 depicts the variation in Ln (*ω_h_*) as a function of 1000/T. This curve exhibits an Arrhenius-type behavior, indicating an activation energy of 0.42 eV. It is noteworthy that this value is very close to the previously determined activation energy for the DC conductivity. This similarity suggests that the mobility of charge carriers in our sample is primarily governed by a simple jump mechanism, with relaxation involving the same charge carriers responsible for conduction. In addition, in the region of higher frequencies and temperatures, the conductivity values are large, attributed to a higher mobility of the charge carriers. We introduce the number of charge carriers n:

σ_dc_ = n e μ (10)

where *σ_dc_* is the DC conductivity, e is the elementary charge, and *μ* is the mobility of the charge carriers. Initially, we considered n as a constant for simplification. However, this assumption is an oversimplification in the context of semiconductors, where the concentration of free carriers can vary significantly with temperature and other factors. In semiconductors, the number of free carriers is often thermally activated, with carriers being excited from defect states or across the band gap. Therefore, n should be expressed as a function of temperature, reflecting these activation processes.

n (T) = n_0_ exp (−2K_B_ T E_g_) (11)

where n_0_ is the effective density of states, E_g_ is the band gap energy, and K_B_ is the Boltzmann constant.

σ_dc_ (T) = n_0_ e μ exp (−2K_B_ T E_g_) (12)

**Figure 10:**
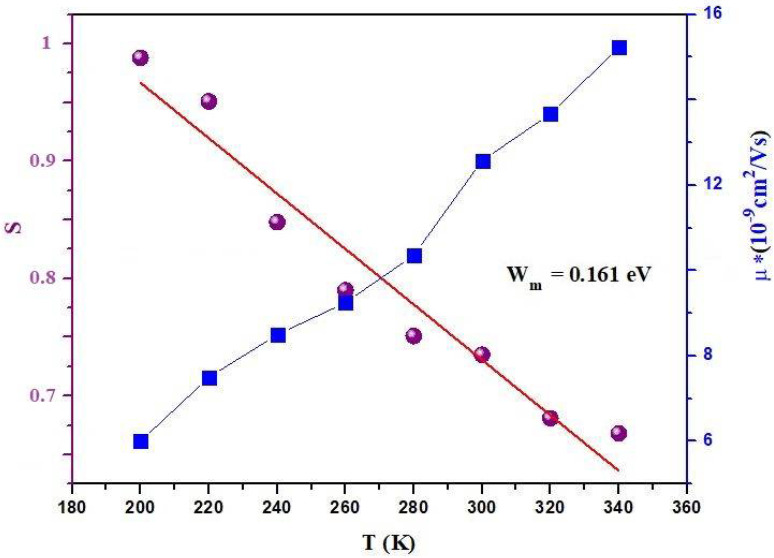
Temperature dependences of the charge carrier mobilities and the exponent S of Bi_0.75_Ba_0.25_(FeMn)_0.5_O_3_ sample.

This revision aligns with the physical reality of semiconductor behavior, where the carrier concentration is not constant but depends on the thermal excitation of carriers. This approach also ensures consistency with our previous discussion regarding the temperature dependence of carrier mobility or diffusion coefficients [32]. Figure 10 illustrates the temperature dependence of the charge carrier mobility for the sample, revealing an increase in mobility with temperature. This motion is less than that observed for electrons and holes [33], except for the electron motion and/or hole motion within the DC conduction mechanism. Our study aims to identify the conduction model that elucidates the electric charge transport phenomena in the studied material.

In our study, we observed a remarkable agreement between the activation energy determined from the DC conductivity and that derived from the jump frequency. This observation can be better understood by examining the relationship between these quantities as described by Equation (9): This relationship indicates that both the DC conductivity and the jump frequency are governed by the same activation energy, When the scaling factor S is close to unity, the activation energies derived from these two parameters should naturally be very similar. This agreement is thus not surprising but rather a confirmation of the underlying physical processes governing the transport mechanisms. Our findings are consistent with this theoretical framework, where the activation energy represents the energy barrier for charge carriers to hop between localized states. The close match of the activation energies derived from different measurements underscores the reliability of our experimental methods and the robustness of our analysis.

To explore the conduction mechanism in our material, various theoretical models have been presented in the literature. These include the correlated barrier hopping model (CBH), where S decreases with rising temperature [34]; the non-overlapping small polaron tunnel (NSPT) model, wherein the exponent s increases with temperature; the quantum mechanical tunnel model (QMT), characterized by an exponent s that exhibits a slight increase with temperature, approaching a value close to 0.8 [35,36,37]; and the overlapping large polaron tunneling (OLPT) model proposed by Long [38], where the exponent S depends on both frequency and temperature, decreasing initially with temperature to a minimum value before increasing again with temperature. The exponent S is plotted as a function of temperature in Figure 10. It becomes evident that the CBH model emerges as the most suitable model to characterize the electrical conduction mechanism in the sample under investigation. If two favorable sites are separated by a distance R, there is a decrease in the barrier height due to the Coulomb interaction from $\omega_{m}$ to W given by:

(13) $$W=\omega_{m}-\frac{ne^{2}}{\pi \varepsilon \left(\omega \right)\varepsilon_{0}R}$$

where W is the barrier height, e is the electronic charge, ε(ω) is the dielectric constant of the material, ε_0_ is the dielectric permittivity of vacuum and n is the number of jumping electrons (polarons) (the density of pair sites is *S* = 1 in the case of a single polaron and *S* = 2 for two polarons). For this model, the parameter s decreases with increasing temperature and it can be expressed as follows [39]:

(14) $$S=1-\frac{6K_{B}T}{\omega_{m+K_{B}Tln\left(\omega \tau_{0}\right)}}$$

where $K_{B}$ the Boltzmann constant is $\omega_{m}$ is the binding energy of the charge carrier. If the ratio *T* is large, the $\omega_{m}$/$K_{B}$ exponent becomes s:

(15) $$s=1+\frac{4K_{B}T}{\omega_{m}}$$

The value of $\omega_{m}$ can be derived from the fit of (Figure 10) using Equation (13). The sample has a binding energy of 0.161 eV.

### 3.4. Dielectric Study

The permittivity, specifically its complex form, is related to the conductivity and impedance. In dielectric materials, the complex permittivity ε* is an important parameter that describes the material’s response to an electric field. The complex permittivity is given by:

ε^∗^ (ω) = ε′(ω) − jε″ (ω) (16)

where ε′(ω) is the real part (dielectric constant) and ε″(ω) is the imaginary part (dielectric loss). The imaginary part ε″(ω) is related to the conductivity σ (ω) through the relation:

ε″(ω) = σ(ω)/ω ε_0_ (17)

This relationship indicates that the dielectric loss is directly proportional to the material’s conductivity. Furthermore, the impedance Z(ω) of the material can be expressed in terms of the complex permittivity:

Z (ω) = 1/jω C_0_ ε^∗^ (ω) (18)

where *C_0_* is the vacuum capacitance of the sample. By combining these equations, we can see that the complex permittivity *ε^∗^ (ω)* is intrinsically linked to the material’s impedance and conductivity and (ω = 2π *f, f* is the frequency).

ε^∗^(ω) = ε′(ω) − j (σ_dc_/ω ε_0_ + _σAC_/ω ε_0_) (19)

Here, *σ_dc_* is the DC conductivity and σacσac represents the AC conductivity contributions. This complex equation demonstrates how both the real and imaginary parts of the permittivity are influenced by the material’s conductive properties.

**Figure 11:**
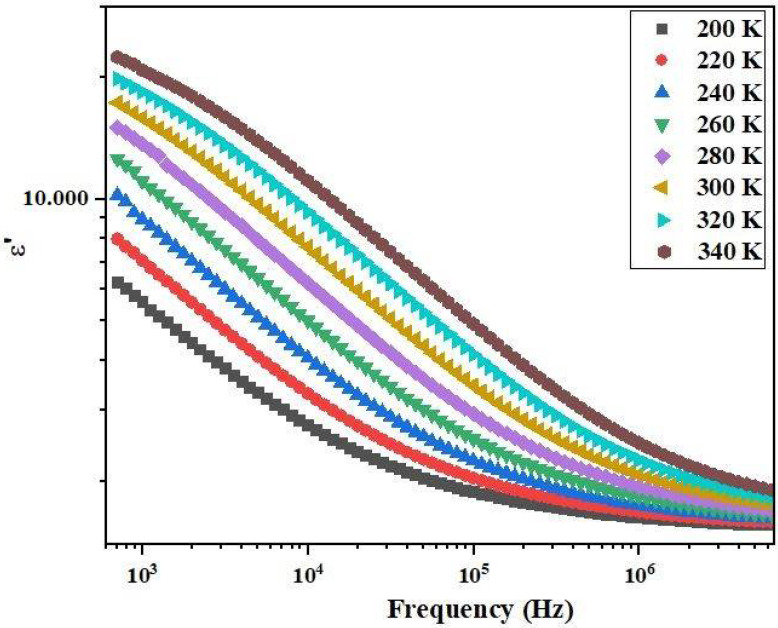
Frequency dependence of the real part of the permittivity (ϵ′) measured using dielectric spectroscopy. At lower frequencies, both σ_AC_ and ϵ′ are influenced by the same physical processes, such as charge carrier mobility and polarization. As frequency increases, σ_AC_ increases due to the enhanced hopping conduction, while ϵ′ decreases due to reduced dipole alignment with the alternating field. The different behaviors highlight the distinction between conduction mechanisms (observed in σ_AC_) and polarization effects (observed in *ϵ′*). By clearly defining the differences in the experimental setups and comparing the frequency dependences, we can provide a comprehensive understanding of the material’s electrical properties. This discussion will elucidate how different measurement techniques probe different aspects of the material behavior under varying frequencies.

Examining the frequency and temperature dependence of the real part of permittivity (*ε′*) in the Bi_0.75_Ba_0.25_(FeMn)_0.5_O_3_ ceramic reveals intriguing insights into its dielectric behavior. As illustrated in Figure 11, there is a discernible increase in ε′ with temperature, particularly notable at frequencies below 10^3^ Hz. In our study, we focus on the limiting frequency of 10^5^ Hz, a characteristic frequency often observed in dielectric studies of perovskites. This frequency is significant as it marks the transition from low-frequency relaxation processes to high-frequency intrinsic dielectric responses. Similar findings are reported by A. Slonopas et al. [40]. This study supports our observation of a limiting frequency, reinforcing the relevance of this parameter in the dielectric behavior of perovskites. In our analysis, we have focused primarily on electronic transport mechanisms, considering the frequency range and temperature conditions relevant to our study. Ionic transport, although significant in perovskites, typically dominates at lower frequencies and higher temperatures, where ion migration can occur more readily. Our chosen frequency range and the experimental conditions were tailored to highlight the electronic contributions to conductivity and dielectric properties. Moreover, ionic transport in perovskites often involves complex interactions and defect states, which require separate in-depth studies. While acknowledging the importance of ionic contributions, we opted to isolate the electronic transport mechanisms to provide a clearer understanding of their behavior under the specified conditions. Future work will aim to integrate the effects of ionic transport, providing a more comprehensive picture of transport phenomena in perovskites.

This augmentation is attributed to the accumulation of charges at the interfaces between the sample and the electrodes. Conversely, at lower frequencies, the real part of permittivity remains relatively high and diminishes with increasing frequency, indicative of dielectric dispersion. This behavior is further elucidated by a polarization process associated with the contribution of grain boundaries. At lower frequencies, charge carriers tend to accumulate in the grain boundary, necessitating more energy for the hopping process, thereby resulting in an augmented dielectric constant [41]. The material exhibits its highest permittivity at room temperature, rendering it advantageous for various electronic applications such as capacitors, transducers, and microwave devices. Its superior dielectric properties make it particularly suitable for energy storage applications, where its ability to store electrical energy efficiently is highly beneficial. Additionally, its responsive dielectric behavior positions it favourably for transducer applications, enabling the efficient conversion between electrical and mechanical energy. Moreover, in microwave applications, the high permittivity of the material allows for effective manipulation of electromagnetic fields, emphasizing its significance in the electronic field.

### 3.5. Thermodynamics Parameters

The determination of thermodynamic parameters such as entropy (*S*), enthalpy (*H*), and free energy of activation (*F*) is crucial for understanding the energetic stability and phase transitions of perovskite materials. These parameters provide insights into the material’s behavior under different conditions, aiding in optimizing its synthesis and performance. By precisely calculating S, H, and F, one can assess the thermodynamic feasibility of various processes involved in the preparation and utilization of perovskite materials, facilitating their application in diverse fields including catalysis, electronics, and energy storage. According to the Eyring theory, the relaxation time can be linked to the free energy through the mobility of charge carriers within the temperature ranges examined [42,43].

(20) $$\tau =(\frac{h}{TK_{B}})\;\exp\;(\frac{\Delta G}{RT})$$

*K_B_* represents Boltzmann’s constant, while R denotes the gas constant, *h* signifies Planck’s constant, and *ΔG* denotes the Gibbs free energy associated with the activation of dipole relaxation during rotation. Furthermore, Δ*G* is interconnected with the activation enthalpy Δ*H* and activation entropy Δ*S* through the following correlation [44]:

(21) ΔG=ΔH−TΔS

(*τ*) Can be formulated as [45]:

(22) $$\tau =(\frac{h}{TK_{B}})\;\exp\;(\frac{\Delta H}{RT})\;\exp\;(\frac{-\Delta S}{R})$$

**Figure 12:**
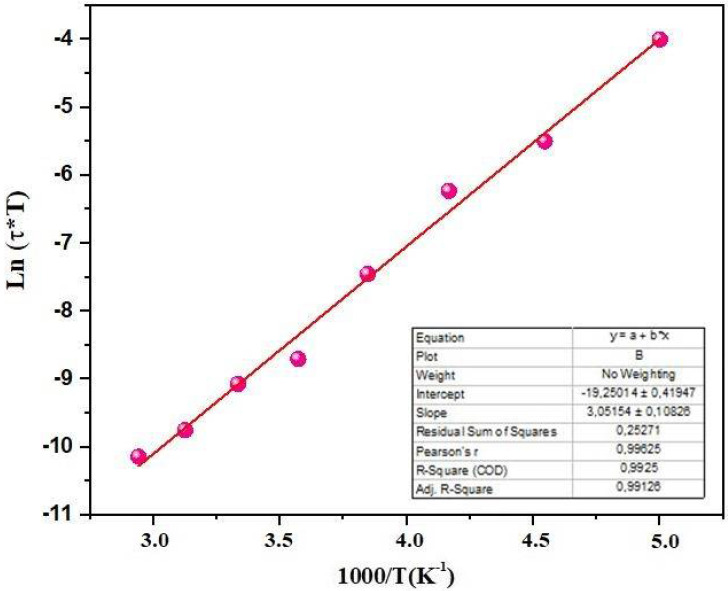
Variation of Ln (τ*T) as a function of 1000/T for Bi_0.75_Ba_0.25_(FeMn)_0.5_O_3_.

The plot of the natural logarithm of (T*τ) against the reciprocal of the temperature depicted in Figure 12 exhibits a straight line, the slope and intercept of which yield the enthalpy and entropy of the system, respectively. The calculated values are *ΔH* = 18.52 kcal/mol and *ΔS* = −2.78 cal/(mol K). The negative value of *ΔS* could arise from dipole-dipole interactions, indicating that molecules tend to be in closer proximity to each other in the activated state [46].

## 4. Conclusions

In summary, the ceramic sample Bi_0.75_Ba_0.25_(FeMn)_0.5_O_3_ was successfully synthesized via the sol-gel route. X-ray diffraction (XRD) analysis confirmed its purity as a single-phase material, devoid of impurities, and assigned it to the rhombohedral R$\overset{´}{3}$C space group. Subsequent SEM and EDX characterizations provided insights into crystallite morphology, average size, and elemental composition. Further analysis using Nyquist plots indicated non-Debye relaxation, with an equivalent circuit model of (R_1_//C_1_//CPE). AC conductivity studies, employing the CBH (Correlated Barrier Hopping) conduction mechanism, were discussed in detail. Activation energy was determined from both DC conductivity measurements. The close agreement of activation energies suggests similarities between the conduction mechanism and relaxation behavior. Additionally, thermodynamic parameters such as enthalpy and entropy were calculated. The dielectric constant and conductivity values imply the material’s potential for various technological applications in the future.

## Figures and Tables

**Table 1:** Structural parameters and angles obtained through refinement of Bi_0.75_Ba_0.25_(FeMn)_0.5_O_3_.

Compound	Bi_0.75_Ba_0.25_(FeMn)_0.5_O_3_
a = b (Å)	5.3986
c (Å)	13.7685
V (Å^3^)	373.326
χ^2^	1.26
R_f_	1.31
R_B_	1.82
α = β (°)	90°
γ (°)	120°
(Bi, Ba) (x, y, z)	(0, 0, 0.248), (0, 0, 0.248)
(Fe, Mn) (x, y, z)	(0, 0, 0), (0, 0, 1/2)
O (x, y, z)	(0.558, 0.987, 0.248)

**Table 2:** Parameters of refinements of the equivalent circuit proposed for the compound Bi_0.75_Ba_0.25_(FeMn)_0.5_O_3_.

T (K)	200	220	240	260	280	300	320
R_1_ (×10^4^ Ω)	460	365	254	233	188	157	123
CPE_1_ (×10^−12^ F)	4.38	4.33	4.32	4.92	5.34	4.71	3.20
C1 (×10^−11^ F)	0.87	0.84	1.12	1.08	1.14	1.26	2.26
Q (×10^−10^ F)	0.62	0.66	0.76	0.85	0.95	1.01	1.09
α	0.647	0.685	0.714	0.733	0.725	0.813	0.837

**Table 3:** Results of adjustment of Jonscher power-law of the alternative conductivity for the compound Bi_0.75_Ba_0.25_(FeMn)_0.5_O_3_.

T (K)	σ (S/m)	A	s
200	2.19 × 10−6	2.13	0.986
220	3.24 × 10−6	1.69	0.953
240	1.52 × 10−5	1.28	0.846
260	4.36 × 10−5	1.25	0.788
280	1.48 × 10−4	0.878	0.752
300	4.33 × 10−4	0.845	0.755
320	2.80 × 10−3	0.721	0.671
340	4.50 × 10−3	0.698	0.667

## Data Availability

The data presented in this study are available from the corresponding author upon reasonable request.
